# HGF percutaneous endocardial injection induces cardiomyocyte proliferation and rescues cardiac function in pigs^☆^

**DOI:** 10.1016/S1674-8301(10)60029-2

**Published:** 2010-05

**Authors:** Zhengxian Tao, Bo Chen, Yingming Zhao, Hongwu Chen, Liansheng Wang, Yonghong Yong, Kejiang Cao, Qifeng Yu, Danian Ke, Hua Wang, Zuze Wu, Zhijian Yang

**Affiliations:** a Department of Cardiology, the First Affiliated Hospital of Nanjing Medical University, Nanjing 210029, Jiangsu Province, China.; b MicroPort Medical (Shanghai) CO., Ltd., Shanghai 201203, China; c Department of Experimental Hematology, Beijing Institute of Radiation Medicine, Beijing 100850, China

**Keywords:** HGF, EnSite NavX, cardiac function, gene therapy, proliferation, myocardial infarction

## Abstract

**Objective:**

To investigate the effect of cardiomyocyte proliferation induced by human hepatocyte growth factor (HGF) in pigs with chronic myocardial infarction (CMI).

**Methods:**

A steerable, deflectable 7F catheter incorporating a 27-guage needle was advanced percutaneously to the left ventricular myocardium of 18 pigs with CMI. Pigs were randomized (1:1:1) to receive adenoviral vector HGF (total dose, 1×10^10^ genome copies), which was administered as five injections into the infarcted myocardium (total, 1.0 mL), or saline, or Ad-null (control groups). Injections were guided by Ensite NavX left ventricular electroanatomical mapping. HGF and cyclin proteins were detected by western blot and immunoprecipitation analysis. Histological and immunohistochemical analysis determined proliferating cardiomyocytes. Myocardial perfusion and cardiac function were estimated by Gated-Single Photon Emission Computed Tomography (G-SPECT).

**Results:**

Western blot analyses showed that HGF were predominantly expressed in the infarct core and border in the myocardium of the infarcted heart. G-SPECT analysis indicated that the HGF group had better cardiac function and myocardial perfusion four weeks after the injection of Ad-HGF than before the injection of Ad-HGF. After treatment there were more proliferating cardiomyocytes in the HGF group compared to either of the control groups. Furthermore, the HGF group myocardial samples expressed higher levels of p-Akt, cyclin A, cyclin E, cyclin D1, cdk2, cdk4 than those in the control groups.

**Conclusion:**

The over-expression of HGF activates pro-survival pathways, induces cardiomyocyte proliferation, and improves the perfusion and function of the porcine CMI heart.

## INTRODUCTION

Human hepatocyte growth factor (HGF), originally identified and cloned as a potent mitogen for hepatocytes[1],[2], has mitogenic, motogenic and morphogenic activities in various cell types via the c-Met/HGF receptor tyrosine kinase. In addition, HGF has cytoprotective and angiogenic activities[3]–[5]. We previously constructed a replication-deficient adenovirus carrying the HGF gene (Ad-HGF)[6]. In experimental animal models, this adenovirus mediates high levels of expression of human HGF, subsequently leading to induction of collateral artery growth and improvement of post-infarct heart function[6],[7]. Preclinical research shows that Ad-HGF is effective in both acute and chronic myocardial ischemic models and no toxicity or mutations have been observed in rats, minipigs or rhesus monkeys[7]–[9]. The clinical application of Ad-HGF has been currently approved by the State Food and Drug Administration (SFDA) of China (No. 2005L01181); the phase I clinical study showed that it is safe and feasible to use an adenovirus gene-transfer vector to deliver the human HGF to individuals with clinically significant coronary artery diseases[10]. Because of its potential angiogenic, antiapoptotic, antifibrotic and stem cell-recruiting effects, HGF has been the subject of increasing attention in cardiovascular diseases[11],[12]. However, the proliferative effect of HGF on cardiomyocytes remains unclear. Our objective was to study the nature of cardiomyocyte proliferation induced by HGF using adenoviral vectors containing HGF and EnSite NavX system-guided injections in pig heart.

## MATERIALS AND METHODS

### Construction of adenoviral vector

Adenoviral vectors constructed for previous experiments were used in this study[6]. HGF cDNA was amplified by polymerase chain reaction using human placenta cDNA library as template. Replication-deficient (E1, E3 deleted) adenoviral vectors containing the human hepatocyte growth factor (Ad-HGF) was constructed by using the pAdEasy-1 system (Stratagene, La Jolla, CA, USA) following the manufacturer's instructions. Construction of replication-deficient adenovirus containing no transgene (Ad-null) or green fluorescent protein (Ad-GFP) was similar to that of Ad-HGF. The recombinant replication defective adenovirus was purified by cesium chloride gradient ultracentrifugation. The final plaque-forming units (pfu) were determined by titration on HEK293 cells under an agarose overlay.

### Chronic ischemic heart model

Eighteen Zhonghua minipigs (male, 3-4 months old, 36±3.4 kg body weight) were housed in the animal care facility of Nanjing Medical University. All animals received humane care in compliance with the Guideline for Care and Use of Laboratory Animals published by Jiangsu Province, China, and approval was granted by Nanjing Medical University ethical review board. Following anesthesia induction by intravenous injection of ketamine (10 mg/kg), the pigs were anesthetized intravenously with 5% barbital sodium. Respiration was maintained through intratracheal intubation by a volume controlled ventilator (Zhongyuan Medical Equipment Inc., Wuxi. China). A thoracotomy incision was made in the fourth intercostal space. The left anterior descending coronary artery (LAD) was exposed and ligated permanently with a 6-0 nonabsorbable surgical suture 1-2 mm below the second diagonal branch to create the myocardial infarction.

### Percutaneous endocardial injection of adenoviral vector

Four weeks after the ligation of the LAD, the animals were randomly divided into three groups (*n* = 6): Ad-HGF injected group (HGF group), Ad-null injected group (Ad-null), and HEPES saline injected group (Saline). The saline and Ad-null treated animals were used as the negative controls. An intramyocardial injection system was used for the percutaneous endocardial injection of adenoviral vector. It consisted of an auto-syringe pump and intramyocardial injection catheter. The auto-syringe pump controlled the injection dose (0.2 ml each time, difference≤1%) and speed (2-25 seconds). The catheter specifications were as follows: sheath compatibility: 7F; usable catheter length: 115 cm; core needle diameter: 27 gauge; core needle “dead space”: 0.9cc; adjustable needle length: 1-7mm; syringe compatibility: 1cc luer lock; curve size: medium and large; Electrode: 4 pcs.

After ≥70 points had been mapped by NavX, the injection catheter was navigated into the infarct region, and five different sites of intramyocardial injections (3 to 5 mm deep, 0.2 ml and 20 seconds each, total volume 1.0 ml, ≥5 mm apart from each other) were performed to the infarct zone. A total of 1×10^10^ genome copies (gcs) of viral vectors in 1.0 ml of HEPES saline (pH 7.4) was used. Identical doses of Ad-null or saline were injected into control animals.

### Cardiac function and myocardial perfusion

Gated-Single Photor Emission Computed Tomography (G-SPECT) was performed in the pigs with a commercially available system (ECAM+; Siemens, Germany). LVEDV, LVESV and LVEF were determined by QGS software. The pigs were habituated to the experimental environment for two hours before the G-SPECT. The imaging was assessed at a dose of 0.3 m Ci/kg of technetium-99m sestamibi (^99m^Tc-MIBI) 4 weeks and 8 weeks after the LAD ligation. Images were acquired 60 to 90 minutes after ^99m^Tc-MIBI injection with a multihead camera with high-resolution collimators. The camera energy window (20%) was set on the 140 ke V photopeak of ^99m^Tc-MIBI. Particular care was taken to avoid pig motion and overlap from extracardiac activity. A total of 32 images (64×64 matrix) were acquired for 40 seconds with 180° rotation. The tomograms were reconstructed in the vertical and horizontal long and short-axis planes.

The myocardial perfusion score was calculated as follows: The left ventricular cavity was divided into 17 segments for assessment of the myocardium[13]. Seven segments were assigned to the LAD distribution area. The five-point scoring used to grade each segment was as follows: 0=absent perfusion; 1=severe hypoperfusion; 2=moderate hypoperfusion; 3=mild hypoperfusion; and 4=normal perfusion. The normal total scores of LAD areas were 28[14].

### Histological and immunohistochemical analysis

The hearts were fixed in 10% formalin, embedded in paraffin and sectioned. Serial sections were made from the apex of the heart to the site of the ligation. Sections were stained with specific antibodies against α-SMA, Ki67 and cardiac sarcomeric α-actinin (Santa Cruz Biotechnology, Santa Cruz, CA, USA) antibodies. Nuclei were stained with 4′,6′-diamino-2-phenilindole (DAPI). Lectin-FITC (Santa Cruz Biotechnology) was used to visualize the blood vessels. The immunohistochemical staining was carried out according to the manufacturer's instructions, and signals were visualized by incubating the sections with Alexa Fluor-488 or -555-labeled secondary antibody (Molecular Probes Inc., Eugene, OR, USA).

For quantification of the vessel-densities in the myocardium, four sections were randomly selected from each group, and six visual fields from each section were observed. As a surrogate for vessel counting, vessel density was determined by lectin optical density measurements using the QWIN100 immunohistochemical imaging analyzer system (Leica Co., Germany).

### Immunoprecipitation and western blot analysis

The tissues were gently homogenized in lysis buffer (50 mM Tris, pH 7.5, 10 mM sodium phosphate, 150 mM NaCl, 1% Triton X-100, 5 mM ethylenediaminetetraacetic acid (EDTA), 10 mM NaF, 5 mM iodoacetate, 1 mM benzamidine, 5 µg/mL leupeptin, 5 µg/mL aprotinin) and incubated on ice for 30 min. The lysates were centrifuged at 10,000 g for 20 min to remove insoluble material and the total protein concentration was determined by a modified Bradford assay. All samples were stored at -80°C prior to electrophoresis. Equal amounts of protein samples (40 µg protein in 20 µL buffer) from each time point were boiled in 3×loading buffer (10 mM Tris-HCl, pH 6.8, including 3% sodium dodecyl sulfate (SDS), 5% β-mercaptoethanol, 20% glycerol and 0.6% bromophenol blue) for 3 min and separated by 10% sodium dodecyl sulfate-polyacrylamide gel electrophoresis (SDS-PAGE) and transferred to a polyvinylidene fluoride (PVDF, Amersham Pharmacia Life Science, USA) membranes. For blocking, membranes were incubated with 5% fat-free milk in Tris-buffered saline at 4°C. The membranes were then incubated with the primary antibodies against human HGF, Akt, p-Akt (Ser473), p21 and p27 (Santa Cruz Biotechnology) for 4h at room temperature. After washing 3 times with PBST, the secondary goat anti-mouse IgG-AP antibody (Santa Cruz Biotechnology) was added for 2 h at room temperature. Finally, the membranes were washed 3 times with PBST, incubated in enhanced chemiluminescence reagents (ECL, Amersham Life Science) for 5 min, and exposed to VA 711 B Blue Sensitive X-ray films. Loading controls of presumably constantly expressed proteins such as GAPDH and Histone 3 (Santa Cruz Biotechnology) were used. Co-immunoprecipitation was performed by incubating the tissues lysates (200 µg) with anti-cdk2 and cdk4 antibody (2 µg/mg of total cell protein) for 16 h at 4 °C, and then adding 20 µl of resuspended volume of Protein G PLUS-Agarose. Tubes were then capped and incubate at 4 °C for 2 h. After centrifugation, the immunoprecipitates were washed in PBST, resuspended in Laemmli buffer, boiled for 5min, loaded onto SDS-PAGE, and transferred to a PVDF membrane. The primary antibody used for immunoblotting were anti-cyclin A, D1, D2, E (1:1,000, Santa Cruz Biotechnology, Inc.), while the secondary antibody was goat anti-mouse IgG-AP (Santa Cruz Biotechnology). The membranes were developed using the ECLplus western blot chemiluminescence detection reagent (Bio-Rad Laboratories, USA), and densitometric analysis was carried out by using image acquisition and analysis software (Scion Image; Scion Corp., USA).

### Statistical Analysis

Statistical analysis was performed with SPSS 10.0 software. Student's t-tests and one-way ANOVA test were used to compare the differences among groups, with statistical significance considered if *P* < 0.05. The data are presented as mean±standard deviation (SD).

## RESULTS

### HGF expression

**Fig. 1:**
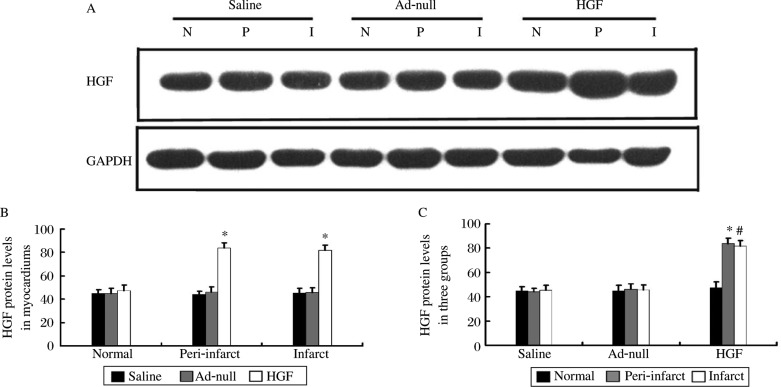
Myocardium HGF expression. A: Representative image of HGF expression. I: infarct zone. P: peri-infarct zone. N: normal zone. B: HGF protein had higher expression in the infarct and peri-infarct zone of the treatment group compared to that in the other zone (*P < 0.01). C: In the HGF group, the myocardial infarct and peri-infarct zone had higher HGF expression than the normal zone (*P < 0.01, ^#^*P* < 0.01).

To investigate whether the adenovirus could mediate HGF expression *in vivo*, western blots were performed four weeks after gene transfer (GTx). There was higher HGF expression in the myocardial infarct and peri-infarct zones of the treatment group than that in the control groups (***Fig.1A and 1B***, *P* < 0.01). In the HGF group, the myocardial infarct and peri-infarct zone had higher HGF expression when compared with the normal zone (***Fig.1A and 1C***, *P* < 0.01), and the normal zone was lowest in each of the three myocardial zones (***Fig.1A and 1C***, *P* < 0.01).These results indicated that the HGF gene expression could be mediated by the adenovirus vector in this porcine chronic myocardical infarction (CMI) model.

### Improvement of myocardial perfusion and cardiac function

**Fig. 2:**
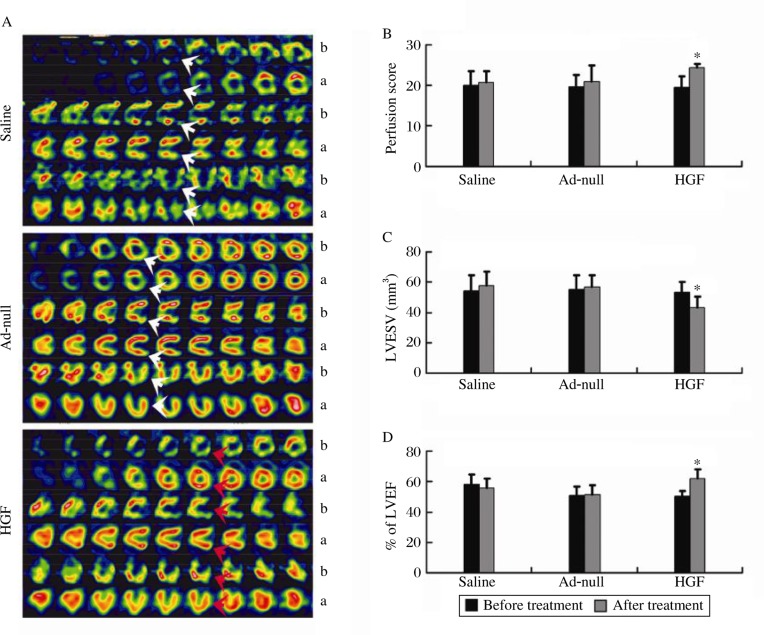
Myocardial perfusion and cardiac function. A: Representative images of G-SPECT. Saline (top), Ad-null (middle) and HGF (bottom) treated porcine chronic infarcted hearts. Red arrows indicate regions with better myocardial perfusion; white arrows indicate regions that had similar myocardial perfusion. b: before GTx; a: four weeks after GTx. B: Bar graph showing myocardial perfusion score, *P = 0.001 *vs* four weeks after vector injection. C: LVEF%, *P < 0.01 *vs* four weeks after vector injection. D: LVESV, *P < 0.01 *vs* four weeks after vector injection.

The myocardial perfusion score in the HGF group was significantly improved four weeks after GTx compared to before GTx (*P* = 0.001, ***Fig. 2A and 2B***). The perfusion scores of Ad-null- and saline-injected pigs four weeks after GTx were similar to the scores before GTx (*P* > 0.05, ***Fig. 2A and 2B***). Identical cardiac function was obtained by G-SPECT. The HGF group had greater LVEF and fewer LVESV four weeks after GTx compared to before GTx (*P* < 0.05, ***Fig. 2A, 2C and 2D***). Thus, the myocardial expression of HGF improved perfusion and rescued cardiac function in the porcine CMI model.

### Cardiomyocyte proliferation

**Fig. 3:**
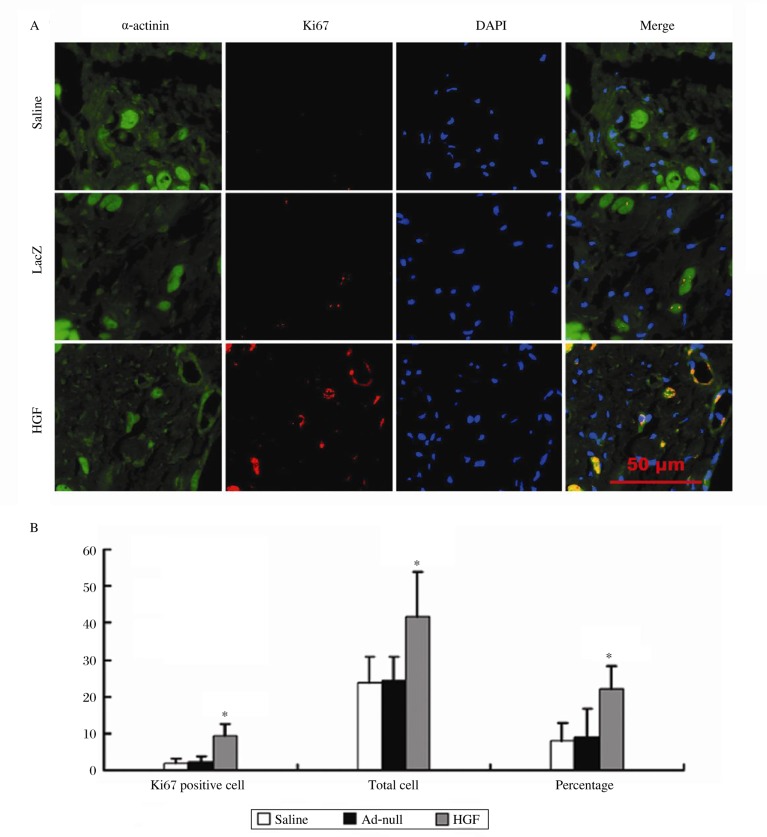
Cardiomyocyte proliferation. A: Representative pictures of sections stained for sarcomeric α-actinin (green) and Ki67 (red). B: Bar graphs showing the quantification of Ki67 positive cardiomyocytes, total cardiomyocytes and the percentage of Ki67 positive cardiomyocytes/ total cardiomyocytes. *P < 0.01 *vs* Ad-null and saline groups.

To determine whether overexpression of HGF increases cardiomyocyte proliferation, we did double labeling using antibodies against Ki67 and sarcomeric α-actinin, a cardiomyocyte-specific marker. As shown in ***Fig. 3A and 3B***, there were more Ki67 positive cardiomyocytes in the HGF-injected hearts (9.20±3.43/mm^2^) than in the saline- (2.00±1.26/mm^2^) and Ad-null- (2.25±1.71/mm^2^) injected hearts (*P* < 0.01). Furthermore, the number of total cells and the percentage of Ki67 positive cardiomyocytes to the total cells were both greater in the HGF-injected hearts (41.60±12.16/mm^2^, 22.29±6.10) compared to the saline- (23.70±7.25/mm^2^, 8.06±4.89) and Ad-null- (24.30±6.49/mm^2^, 9.01±7.59) injected hearts (*P* < 0.01). However, there was no significant difference with either parameter between in the saline- and Ad-null- injected hearts.

### Activation of pro-survival pathway

**Fig. 4:**
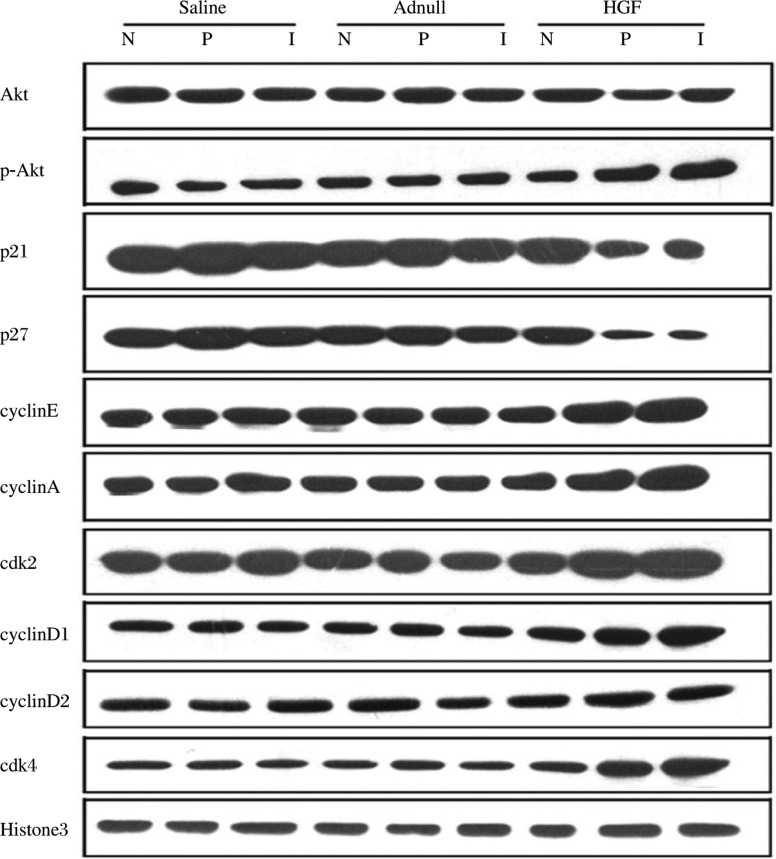
Representative images of western blot (Akt, phosphorylated Akt, p27, p21 and Histone 3) and immunoprecipitations (cyclin D1, cyclin D2, cyclin A, cyclin E, cdk4, cdk2). p27 and p21 were lower in the infarct and peri-infarct zones of HGF group compared to the saline and Ad-null groups (*P* < 0.01). The expression of phosphorylated Akt, cyclin A, E, D1, cdk2 and cdk4 in the infarct and peri-infarct zones of HGF group significantly increased compared to the saline and Ad-null groups. However, there was no significant difference in the expression of cyclin D2 and Akt between the HGF group and control groups. I: infarct zone; P: peri-infarct zone; N: normal zone (*P* > 0.05).

The expression of cell cycle proteins was analyzed to detect the cardiomyocyte proliferation pathway. The expression of phosphorylated Akt was significantly increased in the infarct and peri-infarct zones of the HGF group compared to the saline and Ad-null groups (*P* < 0.01). p27 and p21, cell cycle inhibitors, were lower in the infarct and peri-infarct zones of the HGF group compared to the saline and Ad-null groups (*P* < 0.01). The expression of cyclin D1 in the infarct and peri-infarct zones of the HGF group was significantly increased compare to the saline and Ad-null groups. Similarly, the cdk4 levels in the infarct and peri-infarct zones of the HGF group were significantly greater when compared to values in the saline and Ad-null groups (*P* < 0.01, ***Fig. 4***). However, the expression of cyclin D2 and Akt was not significantly different between the HGF group and control groups (*P* > 0.05). The infarct and peri-infarct zones of the HGF group expressed higher cyclin A and E proteins than those in the saline and Ad-null groups (*P* < 0.01, ***Fig. 4***). The increased cdk2 protein expression in the infarct and peri-infarct zones of the HGF group was also significantly greater than in the saline and Ad-null groups (*P* < 0.01, ***Fig. 4***). Taken together, these results suggested that HGF could increase cardiomyocyte proliferation through the Akt/cyclin D1, A and E pathways.

## DISCUSSION

Heart failure may be the only cardiovascular disease in which the incidence and prevalence rate are increasing. Myocardial infarction is a significant cause of heart failure. It is an enormous challenge to treat postinfarction heart failure, although traditional medication and reperfusion therapy can improve symptoms, no effective therapy currently exists to prevent the progressive decline in cardiac function following a myocardial infarction[11]. Therefore, new therapeutic strategies are needed.

One potential new approach is angiogenic gene transfer. Because of its potential angiogenic, anti-apoptotic, anti-fibrotic and anti-inflammatory benefits, HGF has received increasing attention in ischemic heart disease[10],[11]. However, cardiomyocyte proliferation has not been well studied in hearts treated with angiogenic factors. The capacity of adult cardiomyocytes to re-enter the cell cycle has received considerable attention recently. Since most adult cardiomyocytes are arrested in the G0/G1 phases of the cell cycle[15], investigation has focused on overexpressing the G1-phase-acting cyclins D, B and A[16]–[18]. Although overexpression of these cyclins produced some encouraging results, these studies were based on transgenic technology requiring genetic engineering. Recent evidence also suggested that a population of extracardiac or intracardiac stem cells may be feasible for cardiac repair[19],[20], but this approach has been controversial and requires isolation of autologous stem cells or the use of donor cells along with immunosuppresion. Akt is associated with the promotion of cellular proliferation in many non-cardiac cell types, including oncogenic transformation[21]–[23]. Previous data also demonstrated that Akt kinase expands the population of cycling myocytes in the postnatal heart as well as the number of progenitor cells expressing markers of committed myocyte lineage in the adult heart[24]. Our results showed that HGF overexpression could activate Akt kinase, p21 and p27, induce cyclin D1/cdk4, cyclinE/cdk2 and cyclin A/cdk2 combination, and promote proliferation of cardiomyocytes. Thus, it is possible that HGF treatment *in vivo* might increase proliferation of resident stem cells in the heart or peripheral stem cells recruited to the heart.

For proof of concept, different vector delivery routes have been tested in animal models and clinical trials. Currently, it is unknown which one is the safest and most effective. The effect of intravenous administration of angiogenic growth factors is minimal; this is likely due to “first-pass” uptake by low-affinity receptors in the lungs resulting in considerably lower concentration in the myocardium compared to that with intracoronary administration[25]. Although the use of a minithoracotomy seems to be generally well tolerated, even in patients with advanced myocardial ischemia, nevertheless, the procedure has some risk associated with the administration of general anesthesia and some morbidity associated with surgical manipulation (particularly in patients with previous bypass surgery), and it limits the feasibility of repeat administrations[26]. On the other hand, intracoronary administration of angiogenic growth factors is an attractive modality due to its ease of use and broad applicability. We have used intracoronary injection to deliver Ad-HGF and have had a therapeutic effect in animal experiments and the phase I clinical study. However, in clinical practice, some patients who had complete occlusion in a coronary artery were not allowed to be transplanted with stem cells or HGF via an artery in the infarcted area. Percutaneous catheter-based intramyocardial injection via the NOGA system has previously been used and is an effective approach[27]. However, the NOGA system is very expensive. EnSite NavX is a novel mapping and navigation system that displays the exact anatomic position of conventional catheters in real time. In this study, we developed a set of percutaneous endocardial intramyocardial injectors guided by NavX system to deliver Ad-HGF. This system is protected intellectual property by China (patent number: CN 101536902A). The clinical application of this system has been currently approved by the SFDA of China.

In the HGF group, myocardial infarct and peri-infarct zone had higher HGF expression compared with the normal zone. The normal zone was lowest of the three myocardial zones. The cardiac function and myocardial perfusion scores of the HGF group improved significantly four weeks after GTx compared to those before GTx. These results show that the method of GTx could be effective for individuals with CMI disease.

Although mouse models are invaluable for transgenic and gene knock-out experiments and in the understanding of the biological function of growth factors, the information derived from small animal models may not be directly applicable to human therapy for the following reasons: ① the transduction efficiency of genes is usually much higher in mice than what can be currently achieved in larger animals or humans[28]; ② viral loads and resulting protein concentrations achieved in mouse experiments are not necessarily achievable in larger animals or in humans; ③ the small tissue mass may complicate the determination of both efficacy and correct dose of a growth factor. It is easy to cover the entire myocardium of a mouse with a few injections, while covering the hundreds of times larger human heart is a much more difficult task[29]. An empiric rule of thumb in pharmacology is that drugs need to be tested in at least two animal models, which usually includes a rodent model and a second, non-rodent species. Gene therapy appears to be very vulnerable to misinterpretation if only small animal models are used. The anatomy, physiology and metabolism of pig are analogous to that of a human. Interestingly, the porcine circulation also has three major coronary arteries. Thus, we selected the pig model in this study.

The limitations of this study include: ① A lack of groups that were injected with Ad-HGF via an intracoronary arterial route or surgical epicardial approach individually due to the cost of pig experiments. Thus the effect of the transfer of HGF was not compared to individual transfer. However, our previous published paper indicated a smaller infarct size and better cardiac function via an intracoronary arterial route[7] or surgical epicardial approach[30]; ② Since an Akt inhibitor was not used in this study, there is no direct evidence that the HGF-induced increase of cell cyclin gene expression and cell proliferation was caused by activation of the Akt signaling pathways; and ③ The functions of HGF on induction of endogenous cardiac stem cell homing, proliferation and differentiation were not studied. We will conduct more experiments to address these issues in the future.

We concluded that adenoviral gene transfer of HGF guided by NavX could activate a cardiomyocyte pro-survival pathway, induce cardiomyocyte proliferation and result in the improvement of cardiac function and myocardial perfusion.
